# Adenovirus pneumonia should not be overlooked in immunocompetent youths and adults

**DOI:** 10.1017/S0950268821001485

**Published:** 2021-09-10

**Authors:** Bo Zhao, TengFei Yang, Rui Zheng

**Affiliations:** 1 Department of Pulmonary and Critical Care Medicine, Shengjing Hospital of China Medical University, Shenyang110004, Liaoning Province, China; 2 Department of Health Management & Department of Family Medicine, Shengjing Hospital of China Medical University, Shenyang 110004, Liaoning Province, China

**Keywords:** Adenovirus, immunocompetent youths and adults, pneumonia

## Abstract

Adenovirus pneumonia can occur in immunocompetent youths and adults. We conducted a retrospective analysis on five immunocompetent patients (aged ⩾14 years) with adenovirus pneumonia who visited our fever clinic between 1 February 2020 and 29 February 2020. The symptoms at clinical onset were fever, with cough and phlegm production either absent or appearing several days after disease onset. One patient with severe disease exhibited dyspnoea and a rapid development of respiratory failure. A subset of patients had concurrent gastrointestinal symptoms. The results of blood tests revealed normal leukocyte counts, decreased lymphocyte counts and increased C-reactive protein levels. The imaging findings resembled those of bacterial pneumonia, and pleural effusions were present in some cases. Most patients had a good prognosis with symptomatic treatment and supportive care. However, one patient with severe disease and a MuLBSTA score of >12 had a poor prognosis and ultimately died. Immunocompetent youths and adults may develop adenovirus pneumonia, and severe cases are at the risk of death. Since no effective treatments for adenovirus pneumonia are currently known, the early diagnosis and provision of symptomatic treatment and supportive care should be adopted to prevent the development and progression of severe disease.

## Introduction

Adenoviruses are non-enveloped double-stranded DNA viruses [1]. They can be transmitted via droplets, faeces or contaminated objects, and their common manifestations include upper and lower respiratory tract infections, gastrointestinal symptoms and conjunctivitis [2]. HAdVs are classified into seven species (A–G), including 103 types [3]. Different serotypes can bind to different receptors and lead to different degrees of infection severity [4]. HAdV species B (HAdV-3, 7, 11, 14, 16, 21, 50 and 55), C (HAdV-1, 2, 5 and 6) and E (HAdV-4) are mainly related to respiratory diseases [5]. Adenovirus infections occur throughout the world. Although they are more common in young children, they may also occur in adults who live in crowded and enclosed environments. It is generally believed that adenovirus infections are mild and often self-limiting, and that severe or life-threatening infections mostly occur in infants, young children and immunocompromised patients [4]. Adenoviruses are some of the pathogens that cause community-acquired pneumonia (CAP), and are responsible for approximately 5% of CAP cases in adults [6]. Fatal adenovirus pneumonia rarely occurs in immunocompetent people. However, advances in virus detection techniques in recent years have led to an increased awareness of the importance of respiratory viruses in the aetiology of CAP in youths and adults. In particular, there have been a growing number of outbreaks and sporadic cases of adenovirus pneumonia in immunocompetent patients with the result that patients with severe adenovirus pneumonia have a high fatality rate [6]. We report herein the clinical manifestations and imaging features of youth and adult patients (aged ⩾14 years) with adenovirus pneumonia who visited the fever clinic at our hospital between 1 February 2020 and 29 February 2020.

## Materials and methods

### Study population

**Fig. 1.**
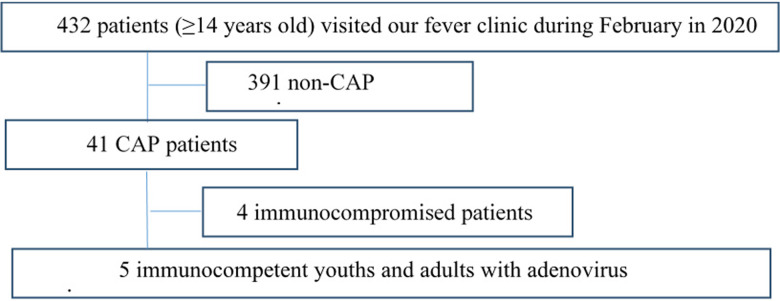
The diagram of the population.

The data from immunocompetent patients with adenovirus pneumonia (aged ⩾14 years) who visited our fever clinic between 1 February 2020 and 29 February 2020 were retrospectively analysed. The diagram of the population is shown in Figure 1. A total of five patients were assessed. The diagnostic criteria for CAP and severe CAP were based on the ‘Guidelines for Diagnosis and Treatment of Community-acquired Pneumonia in Chinese Adults’ issued by the Chinese Thoracic Society in 2016 [7].

As in China, people over 14 years old seek treatment in adult departments. We generally believe that people over 14 are adults, though the guidelines or literature is suitable for people over the age of 18. Because the physical development of adolescents over 14 years old is similar to that of adults, we all follow adult diagnosis standards, but the drug use will meet the requirements of age. Similar situations also occur in different countries [8–10]. The relevant scores below are also used for patients over 14 years old.

### Study methods

Data related to the patients’ general information, clinical symptoms, diagnostic and treatment processes, laboratory testing results, lung imaging results and outcomes were all analysed.

Based on the ‘Guidelines for the diagnosis and treatment of community-acquired pneumonia in Chinese adults’, each patient was assigned a CURB-65 score and a pneumonia severity index (PSI) score. The MuLBSTA score was also calculated for each patient based on the following: multilobular infiltration (5 points); hypo-lymphocytosis ⩽0.8 × 10^9^/l (4 points); bacterial coinfection (4 points); smoking history: acute-smoker (3 points)/quit-smoker (2 points); hypertension (2 points); age ⩾60 years (2 points) [11].

### Statistical analysis

The SPSS17.0 statistical software was used for statistical analysis. Numeric data were expressed as mean ± standard deviation (x̅ ± s), and categorical data were expressed as percentages.

## Results

### General patient information

The five patients with adenovirus pneumonia were all male aged between 16 and 40 years, with a mean age of 26.20 ± 10.26 years. Their mean body mass index (BMI) was 19.79 ± 1.57. The mean maximum body temperature since disease onset was 39.97 ± 0.06 °C.

The medical history of all the patients indicated good health without underlying diseases, such as hypertension, coronary heart disease, diabetes, chronic airway disease and cancer. Two patients had a smoking history, although the number of smoking years and the number of cigarettes smoked were low. All patients had fever at clinical onset, whereas cough and phlegm production were either absent or appeared several days after disease onset. One patient with severe disease exhibited dyspnoea and rapid development of respiratory failure. A subset of patients had fatigue or concurrent gastrointestinal symptoms. The results of blood tests revealed normal leukocyte counts, decreased lymphocyte counts and increased C-reactive protein (CRP) levels (109.12 ± 73.89 mg/l). Procalcitonin levels were below 0.5 ng/ml in most cases. Imaging results resembled those of bacterial pneumonia, which showed predominantly bilateral lung lesions characterised by patchy opacities and consolidation; pleural effusions could be present.

Some patients had a decreased oxygenation index, but dyspnoea symptoms were often not evident. One patient with severe disease received mechanical ventilation. The family refused the use of extracorporeal membrane oxygenation (ECMO), and the patient eventually died. The remaining four patients had mild disease, among which two were hospitalised and two received outpatient treatment; all four patients had a good prognosis. Details are shown in Table 1. The results in Table 1 were taken at admission, and some test results shown in case introductions were taken before they came to our clinic.

**Table 1.** General patient information

	1	2	3	4	5
Age	29	16	16	40	30
Gender	Male	Male	Male	Male	Male
BMI	21.22	18.11	20.05	Unknown	Unknown
Diagnosis	Severe CAP	CAP	CAP	CAP	CAP
Treatment setting	Inpatient	Inpatient	Inpatient	Outpatient	Outpatient
*T*_max_ (number of days from first visit)	40 (8 days)	39.9 (7 days)	40 (6 days)	38.1 (2 days)	Unknown (2 days)
Respiratory tract symptoms (number of days from first visit)	Cough, yellow phlegm (6 days)	Cough, white phlegm (7 days)	Rhinorrhoea (6 days)	Denied	Denied
	Dyspnoea (1 day)		Cough, small amount of yellow phlegm (2 days)		
Other symptoms	Fatigue, diarrhoea	Fatigue	Diarrhoea, nausea, vomiting	Fatigue	None
Medical history	No underlying disease	No underlying disease	No underlying disease	No underlying disease	No underlying disease
Smoking history	Occasional	4 cigarettes/day × 0.5 year	Denied	Denied	Denied
Leukocyte count (3.5–9.5 × 10^9^/l)	5.5	5.8	8.4	9.2	3.5
Lymphocyte count (1.1–2.7 × 10^9^/l)	0.5	0.6	1.3	0.8	0.5
Oxygenation index (mmHg)	124.39	352.38	314.76	–	–
CRP (0–8 mg/l)	218	49.1	129.1	117.2	32.2
Procalcitonin (<0.5 ng/ml)	1.51	0.38	0.403	0.134	0.225
IL-6 (0–7 pg/ml)	214	17.11	–	–	–
SAA (<6.8 mg/l)	426	–	–	–	–
Imaging (days since disease onset)	Multiple patchy opacities in multiple lobes bilaterally, right lower lobe consolidation, small right pleural effusion (6 days)	Right middle lobe inflammation and consolidation, bilateral multilobar inflammation, bilateral (mainly right-sided) pleural effusion (7 days)	Left lower lobe inflammation, with areas of consolidation. Thickened pleura at the left oblique fissure (6 days)	Patchy nodular opacities in the bilateral upper lobes and left lower lobe (2 days)	Patchy opacities in the right middle lobe (2 days)
MuLBSTA score	16	12	0	9	4
CURB-65	1	0	0	0	0
PSI	55	10	15	0	0
Comorbidities	Septic shock, renal insufficiency, abnormal hepatic function, thrombocytopenia, myocardial injury, rhabdomyolysis	None	None	None	None
Prognosis	Deceased	Recovered	Recovered	Recovered	Recovered

SAA, serum amyloid A protein.

### Case 1

The patient was a 29-year-old male. He started to have fever 8 days prior to visiting our clinic. His maximum temperature was 40.0 °C. He experienced fatigue, and he developed cough and yellow phlegm 6 days prior to the visit. He was diagnosed with CAP in a local hospital and was treated with an anti-infective therapy (details unknown), but the symptoms did not subside. The patient exhibited dyspnoea 1 day prior to his visit to our fever clinic.

Lung CT scan findings: multiple patchy opacities in multiple lobes bilaterally, right lower lobe consolidation, small right pleural effusion. Laboratory testing results: leukocyte count 5.5 × 10^9^/l, neutrophil count percentage 88%, neutrophil count 4.9 × 10^9^/l, lymphocyte count 0.5 × 10^9^/l, haemoglobin 149 g/l, platelet count 102 × 10^9^/l, CRP 218.0 mg/l, procalcitonin 1.51 ng/ml. The partial pressure of oxygen was 51 mmHg, with oxygen delivery through a nasal cannula at 5 l/min. Pathogen testing on pharyngeal swabs obtained from the patient was positive for adenovirus DNA and negative for respiratory syncytial virus RNA, influenza A and B nucleic acids, mycoplasma pneumoniae DNA and novel coronavirus nucleic acids. The following tests were also negative: combined antigen/antibody test for HIV, mycoplasma IgM, and blood or sputum bacterial culture.

The patient had severe disease and was admitted to the ward. He did not experience nausea or vomiting since disease onset, but had diarrhoea with loose stools twice a day. Medical history indicated good health without underlying diseases. The patient was allergic to penicillin and cephalosporin. He was an occasional smoker and drinker, with moderate consumption. He denied the following: history of residing in and travelling to COVID-19 outbreak regions, contact with patients with fever and cluster outbreak. He also denied any family history of genetic diseases.

Physical examination on admission: T: 39.3 °C, P: 140 beats/min, R: 45 breaths/min, BP: 134/78 mmHg, arterial oxygen saturation: 84% with oxygen delivery at 10 l/min via face mask. The patient has dyspnoea and was irritable. Harsh breathing sounds were detected in both lungs, and moist rales were detected in the right lung. Physical examination of the heart and abdomen showed no conspicuous abnormalities. The circumferences of the lower extremities were symmetrical, and no oedema was detected. The patient was managed with tracheal intubation, assisted ventilation and sedation. The results of blood gas analysis under the use of ventilator were as follows: pH 7.40, partial pressure of carbon dioxide (pCO_2_) 38 mmHg, partial pressure of oxygen (pO_2_) 44 mmHg, actual bicarbonate (HCO_3_) 23.5 mmol/l, lactic acid (Lac) 1.0 mmol/l, calculated oxygen saturation (SO_2_c) 80%. Although blood or sputum culture was negative, we thought he had bacterial co-infection. So the patient was administered the antibiotics meropenem, moxifloxacin and linezolid, the antivirals ribavirin and arbidol, the immunomodulator thymalfasin, and the anti-inflammatory drug methylprednisolone (40 mg, intravenous administration once a day). Other treatments provided included symptomatic and prophylactic anticoagulation and hepatoprotection. Subsequently, the patient's blood oxygen was difficult to maintain, and the patient exhibited decreased blood pressure, deteriorated hepatic and renal functions, elevated cardiac enzyme levels and rhabdomyolysis. The patient's condition was explained to the family. The family refused ECMO and renal replacement therapy, and the patient died 57 h later.

### Case 2

**Fig. 2.**
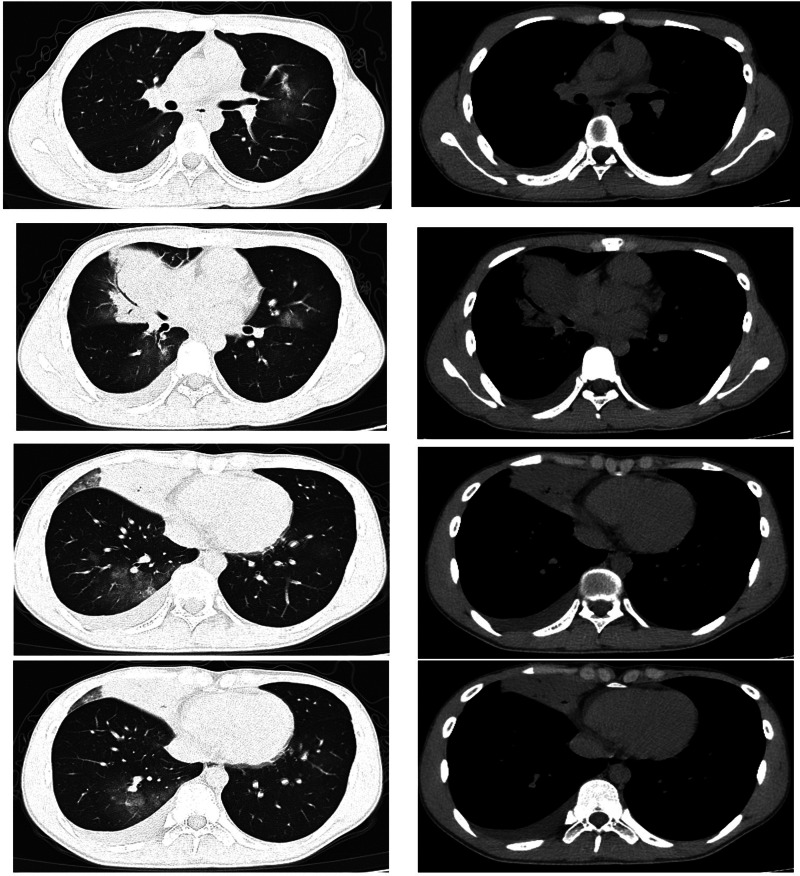
Chest CT of case 2.

The patient was a 16-year-old male. He started to have fever 7 days prior to visiting our clinic. His maximum temperature was 39.9 °C. He also presented with a cough and white phlegm. The patient visited our emergency department on the second day of disease onset. The lung CT scans revealed ‘right middle lobe medial segment inflammation and consolidation’. The results of a routine blood test were: leukocyte count 5.3 × 10^9^/l, neutrophil count percentage 78.6%, neutrophil count 4.17 × 10^9^/l, lymphocyte count 0.65 × 10^9^/l, CRP 21.28 mg/l. The patient's condition did not improve after self-administration of arbidol and cefdinir. The patient was subsequently treated with ceftazidime and an intravenous infusion of azithromycin, but his condition was still not alleviated. A routine follow-up blood test 7 days after disease onset revealed the following: leukocyte count 9.8 × 10^9^/l, neutrophil count percentage 87.6%, neutrophil count 8.54 × 10^9^/l, lymphocyte count 0.86 × 10^9^/l, CRP 82.80 mg/l. The lung CT scans (Fig. 2) showed right middle lobe inflammation and expanded areas of consolidation, new signs of inflammation in multiple lobes bilaterally and bilateral (mainly right-sided) pleural effusion. The patient was admitted to the ward for further treatment.

Since disease onset, the patient had fatigue and no evident dyspnoea and gastrointestinal symptoms. Medical history indicated good health without underlying diseases. He had no history of allergy to medications. The patient smoked four cigarettes/day for 0.5 year, and denied a drinking history. He denied the following: history of residing in and travelling to COVID-19 outbreak regions, contact with patients with fever and cluster outbreak. He also denied a family history of genetic diseases.

Physical examination on admission: T: 37.7 °C, P: 80 beats/min, R: 18 breaths/min, BP: 97/67 mmHg. Harsh breathing sounds were detected in both lungs. No dry or moist rales were heard. Physical examination of the heart and abdomen showed no conspicuous abnormalities. The results of a blood gas analysis without oxygen therapy were as follows: pH 7.46, pCO_2_ 24 mmHg, pO_2_ 74 mmHg, HCO_3_ 6.8 mmol/l, Lac 1.56 mmol/l, SO_2_c 95%. Pathogen testing on pharyngeal swabs obtained from the patient was positive for adenovirus DNA and negative for respiratory syncytial virus RNA, influenza A and B nucleic acids, and novel coronavirus nucleic acids. The combined antigen/antibody test for HIV and blood or sputum bacterial culture was negative. Testing for mycoplasma IgM was positive, but DNA testing for mycoplasma pneumoniae based on pharyngeal swabs was negative. The patient was administered the antiviral ribavirin and the antibiotic azithromycin. He recovered and was discharged after 10 days.

### Case 3

**Fig. 3.**
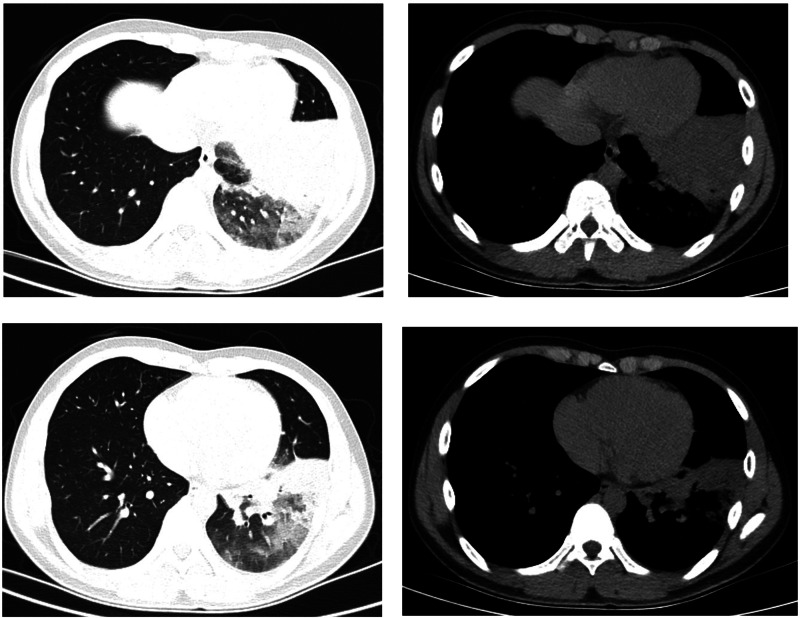
Chest CT of case 3.

The patient was a 16-year-old male. He started to have fever 6 days prior to visiting our clinic. His maximum temperature was 40 °C. He had no respiratory symptoms and no abdominal pain. He exhibited morning diarrhoea with loose stools, accompanied by nausea and vomiting of small amounts of stomach contents. The patient visited our emergency department. The lung CT scans revealed ‘left lower lobe inflammation’. Results of a routine blood test were: leukocyte count 7.0 × 10^9^/l, neutrophil count percentage 73.2%, neutrophil count 5.1 × 10^9^/l, lymphocyte count 1.3 × 10^9^/l, CRP 23.30 mg/l. The patient's condition did not improve after treatment with ceftazidime and intravenous infusion of azithromycin for 3 days. The patient experienced coughing with a small amount of yellow phlegm 2 days ago. The follow-up lung CT scans (Fig. 3) revealed ‘exacerbated left lower lobe inflammation, with areas of consolidation. The pleura was thickened at the left oblique fissure compared with previous scans’. The patient was admitted to the ward for further treatment.

The patient had gastrointestinal symptoms at the early stage of disease. He had no complaint of dyspnoea. Medical history indicated good health without underlying diseases. He had no history of allergy to medications. The patient denied a history of smoking and drinking. He also denied the following: history of residing in and travelling to COVID-19 outbreak regions, contact with patients with fever and cluster outbreak. He also denied a family history of genetic diseases.

Physical examination on admission: T: 40 °C, P: 99 beats/min, R: 20 breaths/min, BP: 110/71 mmHg. Harsh breathing sounds were detected in both lungs. No dry or moist rales were heard. Physical examination of the heart and abdomen showed no conspicuous abnormalities. The results of a blood gas analysis without oxygen therapy were as follows: pH 7.50, pCO_2_ 29.5 mmHg, pO_2_ 66.1 mmHg, HCO_3_ 22.8 mmol/l, SO_2_c 94.7%. Pathogen testing on pharyngeal swabs obtained from the patient was positive for adenovirus DNA and negative for respiratory syncytial virus RNA, influenza A and B nucleic acids, mycoplasma pneumoniae DNA and novel coronavirus nucleic acids. The following tests were also negative: combined antigen/antibody test for HIV, mycoplasma IgM, and blood or sputum bacterial culture. The patient was administered the antiviral arbidol and a combination of antibiotics ertapenem and azithromycin. He recovered and was discharged after 12 days.

### Case 4 and case 5

**Fig. 4.**
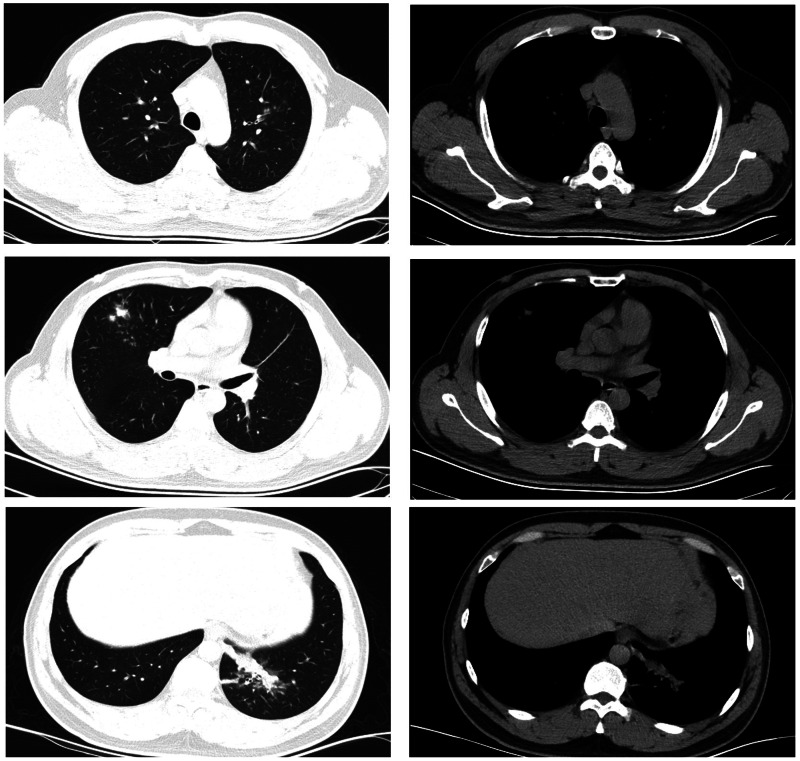
Chest CT of case 4.

**Fig. 5.**
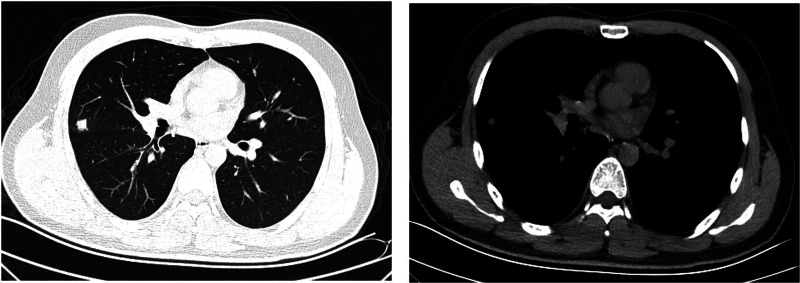
Chest CT of case 5.

Both patients experienced fever with slightly elevated body temperatures. They had no complaints of respiratory symptoms. Since the time of disease onset coincided with the COVID-19 outbreak, the patients visited our fever clinic in order to be screened for COVID-19 infection. Upon examination, both patients were found to have pneumonia (Figs 4 and 5). Pathogen testing on pharyngeal swabs obtained from the patients was positive for adenovirus DNA and negative for the other pathogens tested. Case 4 patient was treated by oral administration of arbidol and intravenous infusion of moxifloxacin for 4 days. Case 5 patient did not receive any special treatment, and subsequently his condition stabilised.

## Discussion

With regard to adenovirus pneumonia, it has previously been believed that the degree of infection severity is mild and often self-limiting, and that severe or life-threatening infections mostly occur in infants, young children and immunocompromised patients. However, there have been a growing number of outbreaks and sporadic cases of adenovirus pneumonia in immunocompetent youths and adults in recent years, with an extremely high mortality rate among patients with severe disease [12, 13]. Due to our insufficient understanding of adenovirus pneumonia and an underestimation of its pathogenicity, few youth or adult patients with CAP receive the relevant testing. When immunocompetent patients develop severe pneumonia, especially the testing results for common pathogens are negative and do not respond to antibiotic treatment, or even rapidly develop respiratory distress and multiple organ failure, adenovirus pneumonia should be considered for the differential diagnosis.

Although adenovirus infections can occur all year-round, most outbreaks occur in winter and early spring [4]. In February 2020 during the COVID-19 outbreak, our hospital established a fever clinic that focused on COVID-19 screening. We actively enhanced the relevant procedures for differential diagnosis, and as a result identified five patients with adenovirus pneumonia. All five of the patients were previously healthy young males, and young men have a higher prevalence which was also mentioned in past studies [9, 14, 15]. They all had fever at disease onset, with a maximum body temperature of up to 40 °C. Cough and phlegm were either absent or appeared a few days after disease onset. A subset of patients had fatigue and concurrent gastrointestinal symptoms. A blood gas analysis was performed for the three inpatients. Two of these had hypoxemia, but had no complaint of dyspnoea. The other patient already exhibited respiratory failure by the time he complained of dyspnoea. His condition deteriorated rapidly, and mechanical ventilation was required. CAP severity is usually scored using CURB-65 and PSI. However, for the patient in the present study who had the most severe disease, the disease severity as assessed by these two scores was mild, indicating they lacked the sensitivity to detect the severity of viral pneumonia. A retrospective clinical study published in 2019 conducted a logistic regression analysis on 528 patients with viral pneumonia and found that the risk factors associated with mortality in patients with viral pneumonia included age ⩾60 years, smoking history, antecedent hypertension, multilobar infiltrates revealed by imaging, lymphocyte count ⩽0.8 × 10^9^/l and bacterial coinfection. All these factors increased the mortality risk of patients with viral pneumonia. Based on these risk factors, a new risk score, named the MuLBSTA score, was established in that study, in which a score of >12 was associated with a high mortality risk for patients with viral pneumonia [11]. The deceased patient in the present study had a high MuLBSTA score of 16 points, indicating that the MuLBSTA score is a better predictor of viral pneumonia compared with other pneumonia scores.

Regarding the results of laboratory tests, most of the patients had normal leukocyte counts, decreased lymphocyte counts, increased CRP levels and procalcitonin levels below 0.5 ng/ml. These results had a considerable resemblance to those in patients with COVID-19. The most common manifestations seen in CT imaging were subpleural and peribronchovascular consolidation with surrounding ground-glass opacities. These findings are more in line with the pathological features of adenovirus pneumonia, which is characterised by bronchial necrosis with haemorrhagic pneumonia in addition to exudative diffuse alveolar damage [16]. The imaging findings of immunocompetent patients with adenovirus pneumonia have been shown to be predominantly unilateral consolidation in the lower lobes, which can be either unifocal or multifocal. It has also been shown that the imaging features of these patients show focal consolidation that can rapidly progress to bilateral consolidation [12, 16]. These imaging findings resemble those of bacterial pneumonia. In addition, pleural effusions may be present. The imaging findings of patients with COVID-19 are characterised by ground-glass opacities and infiltration in the outer third of the lung. Consolidation may be observed in severe cases, and pleural effusion is rare [17]. Thus, adenovirus pneumonia and COVID-19 differ with respect to imaging findings. It is of particular importance not to overlook the possibility of adenovirus pneumonia during the COVID-19 pandemic. Early diagnosis and early provision of symptomatic treatment and supportive care should be adopted to prevent the development and progression of severe disease.

There is currently no effective treatment for adenovirus infections. Some studies have suggested that cidofovir is suitable for treating severe adenovirus infections. However, not all patients require treatment, and the drug is expensive and difficult to obtain [4, 18]. The patient with severe disease in the present study rapidly developed respiratory failure. His condition remained unstable despite tracheal intubation and assisted ventilation. The family refused the use of ECMO, and the patient eventually died. Nonetheless, literature reports show that the overall mortality rate of patients with severe adenovirus pneumonia supported by ECMO remains high [19]. For the patient with severe disease in the present study, a definitive diagnosis was not made at the early stage of the disease. If an early diagnosis of adenovirus pneumonia could have been made, early monitoring of oxygen levels and active supportive care could have been given, which might have improved the prognosis to a certain extent.

## Limitations

Affected by the epidemic situation of SARS-CoV-2 at that time, we had no way to send next generation sequencing for inspection or collect sputum by tracheoscope. The laboratory of our hospital has not yet carried out the determination of adenovirus serotypes. We did some pathogen testing including respiratory syncytial virus RNA, influenza A and B nucleic acids, mycoplasma pneumoniae DNA, novel coronavirus nucleic acids, mycoplasma IgM or bacterial culture. The results of the etiological tests were mentioned in the presentation of each patient's condition. Considering that the patients did not respond to conventional antibiotic treatment and the testing results for common pathogens are negative, we thought they were probably adenovirus pneumonia, though molecular detection of viruses in the nasopharynx and oropharynx does not necessarily indicate causation and could represent infection that is limited to the upper respiratory tract or convalescent phase shedding [20]. The above mentioned is the deficiency of this paper, which needs to be improved in future researches.

## Conclusions

Immunocompetent youths and adults may develop adenovirus pneumonia. Specifically, those with severe disease may even be at the risk of death. They may experience a rapid development of respiratory distress and subsequent multiple organ failure. The following situations should raise the suspicion of adenovirus pneumonia.

1. Patients with fever, respiratory symptoms may be absent at the early stage of the disease, but patients may present with fatigue or gastrointestinal symptoms;
2. Normal leukocyte counts, reduced lymphocyte counts, increased CRP levels, and normal or slightly elevated procalcitonin;
3. Lung imaging resembles that of bacterial pneumonia, the presence of subpleural and peribronchovascular consolidation with surrounding ground-glass opacities, and pleural effusion may be present;
4. No response to conventional antibiotic treatment, and testing results for common pathogens are negative.

Although patients may not exhibit dyspnoea symptoms, they may have hypoxemia. Patients with severe disease, especially those with a MuLBSTA score of >12, may develop respiratory distress and multiple organ failure. These patients are at the risk of death. Early testing and identification of a range of respiratory pathogens, including adenovirus, is important. Since there is currently no effective treatment for adenovirus pneumonia, the early provision of symptomatic treatment and supportive care may prevent the development and progression of severe disease.

## Data Availability

The data analysed during this study are not publicly available due to a need to protect individual's anonymity. These data are confidential, but fully anonymised data may be available from the corresponding author (Rui Zheng; E-mail: zhengr@sj-hospital.org) on reasonable request.
